# Enriching the Mediterranean diet could nourish the brain more effectively

**DOI:** 10.3389/fnut.2024.1489489

**Published:** 2024-11-27

**Authors:** Pasquale Picone, Antonella Girgenti, Miriam Buttacavoli, Domenico Nuzzo

**Affiliations:** ^1^Institute for Biomedical Research and Innovation, National Research Council of Italy, Palermo, Italy; ^2^Department of Biomedicine, Neuroscience and Advanced Diagnostics (Bi.N.D), University of Palermo, Palermo, Italy

**Keywords:** neuroprotection, diet model, Mediterranean lifestyle, brain, food for brain

## Abstract

The increasing prevalence of neurodegenerative disorders represents a challenge to the global health of all nations and populations, particularly with increasing longevity. Urgent prevention strategies are therefore needed, and one opportunity may be to explore the relationship between dietary patterns and brain health which has emerged as a promising strategy. Numerous studies indicate that dietary choices have a significant impact on cognitive function, memory and the risks of neurological disorders, recognizing the dynamic role of diet in maintaining cognitive abilities. One of the most studied dietary styles, the Mediterranean diet, characterized by healthy, plant-based foods fats and moderate consumption of animal products, has demonstrated its neuroprotective potential. Rich in antioxidants, vitamins and polyphenols, this diet shows consistent associations with cardiovascular health and cognitive function. Some less talked about foods, such as seaweed, blackcurrants, Lion’s Mane mushroom and chia seeds, are emerging as potential brain health boosters. These and other new foods could enrich the Western diet making it capable of effectively preventing neurological disorders. Despite promising scientific data, difficulties persist in understanding the complex relationship between nutrition and brain health. Individual variability, long-term dietary adherence, comorbidities, and the need for rigorous clinical evidence pose obstacles. In this review, we would like to focus our attention on the future of brain-diets, which should involve accessible, personalized and evidence-based interventions, providing hope against the challenges posed by neurodegenerative diseases. In fact, as research progresses, more and more attention are being placed to brain health, promising a harmonious and resilient cognitive landscape for individuals and society.

## 1 Introduction

The increasing prevalence of neurodegenerative diseases such as Alzheimer’s, Parkinson’s, and other dementias represents a major global health challenge and as our population ages, there is an urgency to identify effective strategies prevention and intervention are strong. In this context, the interaction between food systems and brain health has emerged as a promising frontier in scientific research (1, 2). In a world where an aging population places an ever-increasing burden on health care systems, recognizing the complex relationship between diet and brain health forces new, accessible approaches promising a comprehensive approach to reducing the onset and progression of neurological diseases. Faced with an aging population and ever-expanding life expectancy, understanding and engaging with the complex processes that regulate brain health is of utmost importance. As our cognitive function, emotional well-being and overall vitality are central and may affect the magnitude of the risk related to neuropathy (3).

In this collaborative review of the relationship between eating and brain health, we delve into the complex scientific data that emphasizes the effects of food on the brain. Understanding the deeper implications of food choices from nutrient dense foods to the potential pitfalls of certain eating behaviors is key to physical health, resilience and a beautiful state of mind promoting improvement, so it is important to unravel the threads that weave nutrition and muscle wellness together. Traditionally, food concepts were significantly associated with physical health and metabolic outcomes. However, a growing body of research indicates that what we eat significantly influences the complex biochemical pathways that govern brain activity (4–6). The brain, the most metabolically active organ, is extremely vulnerable to oxidative stress, inflammation, and the gradual accumulation of damage over time – all factors strongly associated with the development of neurodegenerative diseases (7). Beyond simply providing nutrition, our foods are now recognized as contributing significantly to preserving, and possibly enhancing, cognitive ability. This paradigm shift highlights the need for broader research into food structure and ability a can impact brain health across the lifespan. Historically, the fields of nutrition and neuroscience have served as distinct disciplines, with dietary theory largely tired of addressing physical health problems. But the last few decades have seen a paradigm shift beyond the traditional boundaries separating food and brain. We now appreciate the brain as a metabolic organ, responding in complex ways to the quality and composition of the fuel we provide. The realization that dietary patterns can have profound and lasting effects on cognitive functioning has led to new advances in nutritional neuroscience. Beyond their immediate livelihoods, food choices are widely recognized as craftsmen of long-term brain modification and plasticity Let us dig into the relationship between food patterns and brain health to see how it can promote safety Our insights our grasp of the topic extends beyond a reductive view of nutrients as mere building blocks. Rather, this broad perspective encompasses the complex fabric of interactions between metabolites derived from food and the molecular, cellular, and systemic processes that shape brain function Eating is not like not only as a source of energy but also as a modulator of inflammation, oxidative stress, and cellular repair mechanisms Acknowledge – Factors that play an important role in the pathogenesis and progression of neurological diseases As we begin this review, it is clear that relationships the intricacies of the interplay between food and brain health extend beyond basic nutritional principles. Dietary patterns are now recognized as modulators of inflammation, oxidative stress, and even gut microbiota composition—factors intriguingly associated with the onset and progression of neurodegenerative disorders (8). As we dive into the next sections, we embark on a journey through the growing understanding of the neuroprotective potential of particular food systems, the subtle effects of key nutrients on mood landscapes, food choice and the gut-brain barrier as a complex mediator between. In a world where the demographic pyramid is inverted and age-related neurodegeneration is seen as a dangerous enemy, dietary strategies for strengthening brain health are more than just academic excellence it is a social requirement. The landscape of brain health in the middle you can redefine. In doing so, we contribute to an ongoing narrative that holds the promise of healthy and resilient growth agendas for individuals and nations.

## 2 Mediterranean diet: a blueprint for neuroprotection

**Figure 1:**
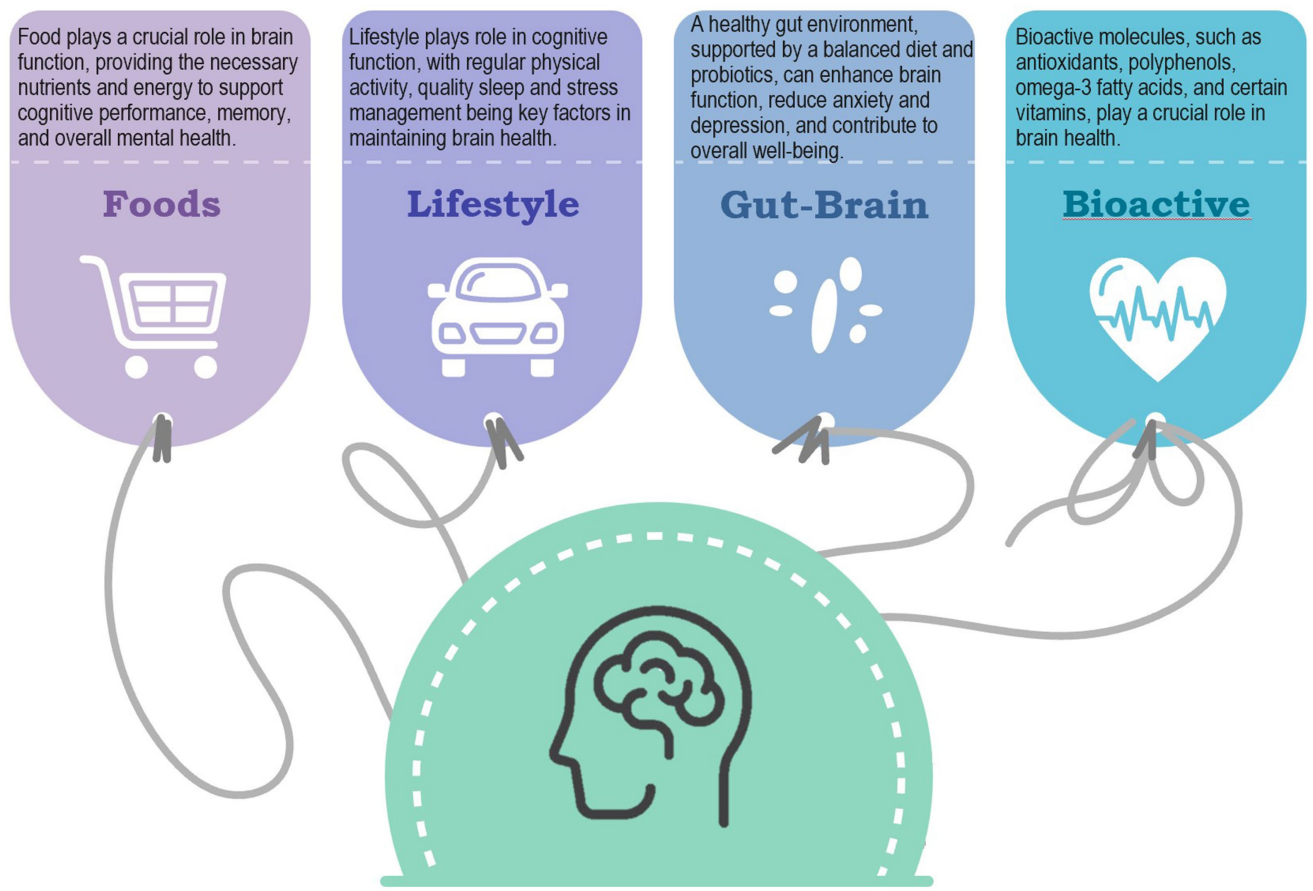
Representative image showcasing key components of the Mediterranean diet and their pivotal role in supporting mental health.

There are numerous diets that show positive effects on health, some of which seem to be closely linked to specific geographical areas. Diets are combinations of foods and beverages consumed by individuals and depend on many cultural, economic and other factors (9). The Mediterranean-style diet, based on the consumption of plant-based foods, healthy fats and moderate use of animal products, has gained wide recognition for its potential health benefits. The Mediterranean lifestyle is based on the traditional Mediterranean diet and includes preparing food, sharing food, harvesting, consuming local and seasonal produce, and frequent socializing with people of all ages and social classes while eating together. This dietary pattern has its roots in the traditional eating patterns of countries bordering the Mediterranean Sea. It continues to be a subject of scientific research today for its association with various health benefits. The Mediterranean diet offers a nutritional model that goes beyond mere sustenance. Scientific studies consistently highlight its potential to promote cardiovascular health, reduce inflammation and even influence cognitive function (Figure 1).

The neuroprotective efficacy of the Mediterranean diet is proven by its rich content of antioxidants, minerals and vitamins. The fruits and vegetables that form the basis of this diet provide a rich source of vitamins (such as vitamins C and E) and polyphenols, which are powerful scavengers of free radicals implicated in neuronal damage (10). The combined effects of these antioxidants go beyond their individual contributions, forming a robust defense against oxidative stress, which is a key factor in the cascade of events leading to neurodegeneration. The following section outlines the key components that make the Mediterranean diet a rich source of antioxidants. Therefore, the most important food-derived molecules for brain health are polyphenols. Infact, they are polyhedral compounds that have been shown to be highly beneficial in counteracting numerous processes including oxidative stress, inflammation and numerous degenerative processes, proving useful in the prevention and treatment of various brain disorders (11, 12) The potential of these compounds for the prevention and treatment of brain disorders is not only related to their ability to reach the brain, depending on their chemical structure, but also to their ability to modulate communication between the brain and the gut, interfering with multiple branches of this axis (12).

Extra virgin olive oil, a key component of the Mediterranean diet, is rich in polyphenols, tocopherols (vitamin E) and carotenoids. These antioxidants have been linked to anti-inflammatory and protective effects on the cardiovascular system (13). Olive oil provides a substantial source of monounsaturated fats, particularly oleic acid. This dietary component has been shown to possess anti-inflammatory properties and is associated with improved cognitive function. Olive oil’s unique combination of antioxidants and beneficial fats contributes to its neuroprotective effects, potentially mitigating the impact of age-related cognitive decline (14).

Furthermore, the daily use of a diverse range of fruit and vegetables, including tomatoes, berries, leafy greens and peppers makes this diet exceptionally capable of maintaining redox homeostasis. Indeed, these foods are rich in vitamins C and E, flavonoids and carotenoids and offer a diverse range of antioxidants (10).

Nuts and seeds, especially walnuts and almonds, are rich in antioxidants, including vitamin E and polyphenols. These foods contribute not only to the antioxidant profile, but also to heart-healthy fats in the diet (15). Nuts and seeds are rich in essential nutrients and bioactive compounds and, consequently, have attracted scientific interest as a potential source of neuroprotective agents (16). These small but dense nutritional powerhouses provide a variety of elements that support brain health and may play a role in the prevention of neurodegenerative diseases. Magnesium, found in many nuts and seeds, is essential for nerve function and the transmission of nerve signals. Optimal levels of magnesium are associated with maintaining a healthy nervous system, which in turn can reduce the risk of neurodegenerative diseases (17). Several nuts and seeds contain amino acids that act as precursors of neurotransmitters, the brain’s chemical messengers. For example, sunflower seeds contain tryptophan, a precursor of serotonin, which plays a role in mood regulation and may contribute to mental well-being (18).

The habitual use of herbs and spices, including oregano, thyme, rosemary and turmeric, enhances the flavor and antioxidant content of Mediterranean cuisine. For example, oregano is particularly rich in rosmarinic acid, a powerful antioxidant (19). Preclinical studies have shown that rosemary extracts have neuroprotective effects. The active compounds in rosemary have been shown to help protect neurons from damage, improve neuron survival and promote overall brain health. These effects are particularly relevant in the context of neurodegenerative disorders such as Alzheimer’s and Parkinson’s disease (20, 21). Preclinical studies have shown that rosemary extracts have neuroprotective effects. The active compounds in rosemary have also been shown to protect neurons from damage, increase neuron survival and promote overall brain health (22).

Oily fish, such as sardines and mackerel, are a key component of the Mediterranean diet and provide omega-3 fatty acids as well as selenium and astaxanthin, powerful antioxidants with anti-inflammatory properties (23). It is impossible to overestimate the neuroprotective potential of omega-3 fatty acids, especially docosahexaenoic acid (DHA) and eicosapentaenoic acid (EPA). These essential fatty acids are integral to maintaining the structural integrity of neuronal membranes, modulating inflammation and promoting synaptic plasticity, all vital aspects of brain health and resilience (24). In this perspective, Godos and colleagues in a meta-analysis suggested that fish consumption is associated with a lower risk of cognitive impairment/decline in a dose–response manner (25). Considering that in the style of this diet physical activity and sport play an important role, it is worth underlining that omega-3 could play a beneficial role in this aspect as well (26).

Whole grains, including brown rice, quinoa and whole wheat, are a source of antioxidants such as ferulic acid and lignans, which contribute to the overall antioxidant richness of the diet (27). Whole grains, the main source of carbohydrates in the Mediterranean diet, contribute to the neuroprotective milieu by modulating glucose metabolism. Whole grains have a lower glycemic index than refined grains, resulting in sustained glucose release and the promotion of stable blood sugar levels. This controlled glucose metabolism is of great importance for the prevention of insulin resistance, which is a factor linked to cognitive decline and neurodegenerative disorders (7, 28). In addition to its nutritional components, the Mediterranean diet is also significant for the social and cultural aspects of eating. The convivial nature of shared meals, the variety of flavors and the incorporation of seasonal and locally sourced ingredients contribute to an overall approach to wellbeing characterized by a focus on the social and cultural aspects of eating. These aspects may also exert an indirect influence on stress levels and mental health, thus enhancing the neuroprotective potential of diet. These food components, which greatly benefit brain health, include vitamins, minerals, fatty acids, carotenoids, polyphenols, bioactive peptides, probiotics, creatine, and saponins (16, 29–31). A substantial body of epidemiological and clinical evidence has linked adherence to this dietary pattern with a reduction in the risk of cognitive decline, Alzheimer’s disease and other neurodegenerative disorders. These results highlight the practical relevance of adopting a Mediterranean-style diet for brain health in different populations. The intersection between diet and neurology has become a focal point of scientific investigation and, within this landscape, the Mediterranean diet has emerged as a topic of considerable interest for further investigation. Epidemiological studies analyzing the patterns, distribution and determinants of health and disease in populations have provided a substantial body of evidence supporting the idea that the Mediterranean diet exerts profound neuroprotective benefits (32).

Thus, the Mediterranean diet represents a paradigm for neuroprotection, integrating a complex network of antioxidant, anti-inflammatory, neurotrophic and socio-cultural components. Its comprehensive approach, which includes not only specific nutrients but also social and lifestyle factors, positions it as a highly effective ally in the quest for cognitive well-being and the prevention of neurodegenerative disorders.

## 3 Potential mechanisms linking the Mediterranean diet to brain function

Numerous observational studies had already indicated that specific foods or nutrients such as fish, unsaturated fatty acids, antioxidants, vitamins folates, carotenes and flavonoids may have a potential protective effect against dementia or cognitive decline (4). Unfortunately, all these data on isolated nutrients or foods so far seem conflicting. Compared to studies based on single nutrients, they ignore important interactions (additive, synergistic or antagonistic effects) between dietary components and, above all, people do not eat isolated nutrients (33). Therefore, studies based on the overall analysis of foods could explain the biological mechanisms for understanding the association between foods or nutrients in the Mediterranean diet as a potential for better brain health. Indeed, the role of nutrition and in particular the Mediterranean diet on cardiovascular events is well documented (34, 35) and a protective effect on brain disease should also be expected.

There are various mechanisms by which nutrition can influence the brain and prevent or slow down neuronal damage, among these we can remember the correct intake of minerals and vitamins cofactors of numerous biological brain processes (36). But the mechanisms most implicated in the process of neurodegeneration are oxidative damage implicated in the pathogenesis of AD and other neurological diseases, the Mediterranean diet is also a dietary model with antioxidant properties (37). Furthermore, inflammation is another mechanism involved in the pathogenesis of neurodegenerative diseases that, in general, is reduced with greater adherence to the Mediterranean diet (37).

Recent data indicate that the Mediterranean diet exerts beneficial effects on gene expression through epigenetic modifications, offering protection against chronic diseases and inflammation. The data highlight how key components of the Mediterranean diet, such as polyphenols, monounsaturated fatty acids, and phytonutrients from fruits and olive oil, modulate gene expression through DNA methylation and histone modification. These epigenetic mechanisms reduce inflammation and positively influence metabolic pathways related to disease prevention, highlighting the role of the diet in supporting long-term health (20).

The Mediterranean diet also supports anti-aging processes through specific epigenetic mechanisms, as highlighted by Calcaterra et al. Key bioactive compounds, such as polyphenols, omega-3 fatty acids, and micronutrients, contribute to DNA methylation, histone modification, and regulation of non-coding RNAs, which are critical for modulating gene expression. These compounds work to reduce pro-inflammatory genes and increase antioxidant pathways, effectively reducing inflammaging. By acting on pathways related to cellular stress response, apoptosis, and immune regulation, the Mediterranean diet demonstrates a broad epigenetic influence that promotes healthy aging and chronic disease prevention (38).

An interesting and increasingly recognized mechanism in the scientific community is the role of nutrition in maintaining and enhancing microbiota, which has a beneficial impact on both nervous and psychological functions. The gut-brain axis, a bidirectional communication network between the gut and the brain, plays a fundamental role in this interaction. A well-balanced microbiota is essential to produce neuromodulatory molecules, such as serotonin and short-chain fatty acids, which directly influence mood, cognitive function and emotional well-being (39). Numerous studies have shown that typical foods of the Mediterranean diet, rich in fiber, polyphenols, omega-3 fatty acids and fermented products, promote intestinal homeostasis. These dietary elements support the growth of beneficial bacteria, reduce inflammation and promote the production of metabolites that act as signaling molecules, helping to improve intestinal health and, consequently, brain function (40).

## 4 Interactions between dietary components and their impact on brain health

Several studies suggest that the impact of dietary patterns on brain health is not attributable solely to single nutrients, but rather to complex interactions between multiple dietary components. These interactions can modulate the bioavailability, metabolism, and bioactivity of nutrients, influencing their neuroprotective potential. For example, the combination of polyphenols from fruits and vegetables with omega-3 fatty acids, commonly present in the Mediterranean diet, has been shown to exert synergistic effects, improving antioxidant capacity and reducing neuroinflammation (41).

In addition, dietary fiber plays a crucial role in modulating gut-brain communication, as it influences the production of short-chain fatty acids (SCFA) by the gut microbiota. These metabolites have been implicated in modulating neuroinflammatory pathways and maintaining the integrity of the blood–brain barrier (42). When fiber intake is combined with polyphenol-rich foods, such as berries, the bioavailability and activity of these compounds can be improved due to their interaction with the gut microbiota, leading to a more significant effect on cognitive function. Furthermore, some fibers, such as pectin, appear to have effects on neurons (43).

The combination of dietary components also affects the regulation of neurotransmitter synthesis. For example, the presence of certain amino acids, such as tryptophan, together with antioxidant-rich foods can support serotonin production and reduce oxidative stress in neural tissues (44). This interaction suggests that combinations of nutrients may have a more substantial effect on mood and cognitive function than single nutrients alone.

## 5 Food emerges as a support to the Mediterranean diet for brain health

Certain foods are emerging as potential supporters of brain health. In fact, the Mediterranean diet is being enriched with foods not typically found in the Mediterranean basin and together with an increased awareness of the population, this diet is gradually becoming more robust.

This paradigm shift emphasizes the need for a holistic approach to health, in which nutritional choices are fundamental to cognitive function. The idea that food can serve to promote brain health is consistent with the growing body of evidence emphasizing the complex interconnection between nutrition and cognitive well-being. The realization that food choices can impact brain function and may have long-term implications for neurological health is a significant and evolving aspect of contemporary scientific understanding.

The following section will describe the dietary habits of the Mediterranean population, with a particular focus on foods that are becoming increasingly prevalent in their diets. These foods present a potential avenue for enhancing the diet with novel health elements that may also exert beneficial effects on cognitive function.

### 5.1 Seaweed

The term “seaweed” is used to describe a diverse range of marine algae that thrive in oceanic environments. These organisms are not true plants, but are simple, photosynthetic organisms that play a crucial role in marine ecosystems. Seaweeds can be classified into three principal groups on the basis of their color: red, green, and brown algae. Red algae are typically found in deeper waters and are characterized by the presence of red pigments. Notable examples include dulse and nori, which is a common ingredient in sushi. Green algae are typically found in shallow waters and often exhibit pigmentation patterns like those observed in terrestrial plants. An exemplary of this category is sea lettuce. Brown algae are typically found in colder waters. They can reach considerable size and are known for their brownish color. Notable examples include kelp, which can form dense underwater forests. The nutrient content of seaweed may contribute to brain health. Although the direct impact of seaweed on the brain may not have been as extensively studied as that of other foods, it offers several elements that can contribute to overall cognitive well-being. Some varieties of seaweed, including algae, are notable for their high omega-3 fatty acid content. These fatty acids, particularly docosahexaenoic acid (DHA), are essential for optimal brain health and are known to support cognitive functions (45). Additionally, seaweed is a rich source of iodine, a vital mineral for the optimal functioning of the thyroid gland. The thyroid gland is responsible for the production of hormones that are integral to brain development and function. Therefore, it is crucial to maintain optimal levels of iodine to ensure cognitive health. Seaweed contains a variety of vitamins and minerals, including vitamin B12, vitamin C, calcium, and iron, which are vital for overall health and cognitive function. Some seaweeds contain antioxidants that can help protect the brain from oxidative stress, which is associated with aging and certain neurodegenerative conditions (46). It is important to note that while seaweed can be a nutritious addition to a balanced diet, excessive consumption should be avoided. Some types of seaweed can be high in iodine, and an excessive intake of iodine may have adverse effects on thyroid function.

### 5.2 Blackcurrant

Blackcurrants are rich in various nutrients and compounds that can contribute to overall health, including brain health. Blackcurrants are high in antioxidants, including anthocyanins, flavonoids, and vitamin C (47) which is not only a powerful antioxidant but also plays a role in collagen synthesis. Collagen is essential for the health of blood vessels, including those in the brain. The deep purple color of blackcurrants is due to the presence of anthocyanins, which have been linked to improved cognitive function. Research suggests that anthocyanins may help protect the brain from inflammation and oxidative stress (48). Blackcurrants contain polyphenolic compounds, which have been associated with various health benefits, including anti-inflammatory effects. Chronic inflammation is implicated in several neurodegenerative diseases, so consuming foods rich in polyphenols may support brain health. Blackcurrants contain vitamin K, which is important for maintaining brain health (10). Vitamin K is involved in the synthesis of sphingolipids, a type of lipid found in high concentrations in the brain.

### 5.3 Lion’s mane mushroom

Lion’s Mane Mushroom (Hericium erinaceus) is a type of mushroom that has been employed in certain traditional medical practices, particularly in Asian cultures. It has attracted attention for its potential health benefits, including those that may be related to brain health. While research is ongoing, some studies and anecdotal evidence suggest several potential associations between lion’s mane mushroom and brain health. The Lion’s Mane Mushroom contains compounds that have been demonstrated to stimulate the production of Nerve Growth Factor (NGF) (49). Nerve growth factor (NGF) is a protein that plays a pivotal role in the growth, maintenance, and survival of nerve cells, thereby potentially supporting brain health. A number of studies, primarily conducted in animal models, have indicated that lion’s mane mushroom may have cognitive-enhancing effects. These effects may be related to the mushroom’s potential to support neurogenesis (the growth and development of neurons) and protect against neuronal damage (50). The Lion’s Mane Mushroom displays antioxidant and anti-inflammatory properties, which are vital for the protection of the brain from oxidative stress and inflammation. Chronic inflammation and oxidative stress have been linked to a number of neurological disorders (51). The available evidence suggests that Lion’s Mane Mushroom may have a beneficial effect on memory and learning abilities. Nevertheless, further research, particularly in human subjects, is required to establish a definitive correlation.

### 5.4 Chia seeds

Chia seeds are minute black or white seeds that originate from the *Salvia hispanica* plant, which is indigenous to regions of Central and South America. The seeds have gained popularity as a nutritious addition to various diets due to their rich nutritional profile. Although research specifically examining the impact of chia seeds on the brain is limited, their overall nutritional content may contribute to brain health in several ways. In the APP23 model of Alzheimer’s disease, it was observed that chia seeds were capable of enhancing spatial learning deficits without negatively affecting cognitive flexibility. This improvement is likely associated with enhanced glucose tolerance, reduced corticosterone levels, and the reversal of the SRD-induced rise in proinflammatory cytokine levels (52). Chia seeds are a plant-based source of alpha-linolenic acid (ALA), which is a type of omega-3 fatty acid. Omega-3 fatty acids are essential for optimal brain health. While ALA is not as potent as the omega-3 s found in fish (EPA and DHA), it still plays a role in supporting overall well-being. Chia seeds contain a variety of antioxidants, including chlorogenic acid and quercetin. Antioxidants assist in the protection of the brain from oxidative stress, which has been demonstrated to contribute to the aging process and the development of neurodegenerative diseases (53). Chia seeds are a rich source of dietary fiber. A healthy digestive system is linked to better brain health. Some research suggests that gut health may have indirect effects on brain function through the gut-brain axis (54). Chia seeds are a relatively rich source of protein and also provide a number of essential nutrients, including calcium, magnesium and phosphorus. These nutrients are vital for overall health, including that of the brain.

## 6 Challenges and future directions

It is critical to recognize the importance of nutrition to the brain and its influence on cognitive function. While nutritional neuroscience research continues to yield significant insights, the role of marketing in the brain health space is becoming increasingly critical. Despite advances in food quality and a more complete understanding of the nutritional needs of the human body, a knowledge gap persists regarding the precise nutritional requirements of the brain. One of the major challenges in clarifying the relationship between nutrition and brain health is that there is an intricate interplay of cause and effect, which occurs over time. The brain is a metabolically active organ that requires a constant supply of nutrients to maintain optimal functioning. It is therefore reasonable to conclude that fueling the brain is important at all stages of life. However, providing healthy foods does not necessarily guarantee successful brain development or maintenance of optimal brain function into adulthood. It is now well established that the number of neurons in the brain is established at birth, with the vast majority of these cells present from the moment of conception. However, the development of synapses, or connections between neurons, continues throughout the first 2 years of life. It is important to note that while some foods and dietary patterns have been shown to have beneficial effects, no single food can guarantee optimal brain health. Lifestyle factors, including physical activity, adequate sleep, stress management, and overall mental well-being, also play a significant role in cognitive function.

One of the primary challenges lies in the considerable variability observed in individual responses to dietary interventions. Many factors contribute to this complexity, including genetic predisposition, composition of the gut microbiota, and lifestyle variables. It is therefore essential to design personalized dietary strategies that account for these individual differences to optimize outcomes and ensure the practical applicability of dietary recommendations. The sustainability of dietary patterns represents a significant challenge. Long-term adherence to dietary regimens may prove challenging for many individuals due to various factors, including lifestyle, taste preferences and social dynamics. It is therefore essential to identify ways of enhancing adherence, whether through modified dietary approaches or behavioral interventions, to realize the potential long-term benefits. Although preclinical studies and observational data offer valuable insights, the translation of these findings into evidence-based dietary recommendations necessitates the implementation of rigorous clinical trials. It is imperative that well-designed, long-term studies that incorporate diverse populations, consider confounding variables, and employ standardized methodologies are conducted. Such trials are vital for establishing causal relationships, determining optimal dietary compositions, and addressing the limitations of current research. The combined and interactive effects of different dietary components present a complex challenge to researchers. It is of the utmost importance to gain an understanding of how different nutrients, bioactive compounds and dietary patterns interact with one another within the complex milieu of brain health. An investigation into these interactions will facilitate the formulation of comprehensive dietary recommendations that capitalize on the collective benefits of diverse dietary components. It is of the utmost importance to advance our mechanistic understanding of how specific dietary components exert their effects on the brain. A deeper comprehension will be achieved through the elucidation of the cellular and molecular pathways through which nutrients, bioactive compounds, and dietary patterns influence neuroprotection. This knowledge is fundamental to the development of targeted interventions and pharmacological strategies that mimic the beneficial effects of certain dietary elements. The possibility of tailoring dietary interventions to specific neurodegenerative diseases represents a promising avenue for further investigation. The pathological mechanisms underlying different conditions, such as Alzheimer’s, Parkinson’s, and Huntington’s diseases, are distinct. The design of dietary strategies that target disease-specific vulnerabilities and mechanisms offers a promising avenue for precision medicine approaches in the prevention and management of neurodegenerative disorders. It is of paramount importance to ensure that neuroprotective dietary strategies are made accessible to diverse populations. It is of the utmost importance to address the issues of affordability, food availability and cultural acceptability in order to ensure the success of public health initiatives. The integration of dietary recommendations into existing public health frameworks will enhance the reach and impact of these interventions, as well as facilitate the development of strategies that are more effective in promoting healthy eating habits. The relationship between diet and brain health is a complex and evolving field of study. It is imperative to address the challenges posed by individual variability, long-term adherence, and the necessity for rigorous clinical evidence. As research progresses, a sophisticated comprehension of the impact of diet on brain health will inform the formulation of bespoke, culturally appropriate and evidence-based strategies for the prevention and management of neurodegenerative diseases. It is anticipated that interdisciplinary collaboration between nutritionists, neuroscientists, clinicians and public health experts will prove pivotal in determining the future direction of this field.

## 7 Conclusion

In the complex relationship between diet and brain health, our research in the fields of neuroscience and nutrition has revealed a sophisticated web of influences that extend far beyond the mere sustenance of the body. As we traverse this expansive landscape, it becomes evident that dietary choices exert a profound impact on the molecular, cellular, and systemic processes that underpin cognitive function. From the rich and diverse antioxidants present in the Mediterranean diet to the neurotrophic effects of omega-3 fatty acids, each nutrient and dietary pattern contributes to a complex interplay of effects that collectively fortify the brain against the ravages of neurodegeneration. The Mediterranean diet, which has been lauded as a model for neuroprotection, incorporates a variety of antioxidants, healthy fats, and culinary diversity. This dietary paradigm challenges the reductionist view of nutrients as mere fuel, instead positioning them as sculptors of long-term resilience. In addition to providing immediate sustenance, the Mediterranean diet nurtures the brain through the antioxidative properties of fruits and vegetables, the omega-3 fatty acids found in fatty fish, the stable glycemic index of whole grains, and the neuroprotective effects of olive oil. It is not merely a nutritional guide; rather, it is a cultural and lifestyle compass that underscores the holistic nature of well-being. Omega-3 fatty acids, as key nutrients for brain nourishment, have been demonstrated to extend their reach from providing structural foundations to offering neurotrophic support. Their pivotal role in maintaining neuronal membrane integrity, modulating inflammation and supporting synaptic plasticity renders them indispensable to cognitive resilience. In addition to providing nutrients, marine sources such as omega-3 fatty acids also offer protection against cognitive decline. The domain of nutrients as neuroprotectors is vast and encompasses a coalition of antioxidants, vitamin D, and other compounds that collectively combat oxidative stress and inflammation. Antioxidants, in the form of vitamins C and E, act as sentinels against free radicals. Vitamin D, which has been traditionally associated with bone health, has been shown to possess anti-inflammatory and immunomodulatory properties. The B vitamin group is responsible for regulating homocysteine, while zinc and selenium act as trace element allies. Polyphenols, meanwhile, function as modulators of inflammation and oxidative stress. In this symphony, each nutrient performs a specific yet interrelated function in maintaining the brain’s vitality. As we stand at this juncture of synthesis, we are confronted with a number of challenges and must consider future directions. The interplay of dietary factors on brain health is a complex phenomenon, and our understanding of this intricate process must transcend reductionist approaches. Individual variability necessitates the implementation of personalized approaches, long-term adherence requires the development of innovative strategies, and the necessity for rigorous clinical trials demands a commitment to evidence-based practice. The interactions between dietary components, ethical considerations and cultural sensitivity highlight the need for a holistic and inclusive approach. The vision for the future encompasses a comprehensive understanding of dietary influences across the lifespan, with lifecourse perspectives, disease-specific approaches and public health integration representing integral components of this narrative. As research unveils the mechanistic intricacies of dietary effects on the brain, the promise of accessible, personalized, and evidence-based interventions emerges as a beacon of hope amidst the challenges.

In conclusion, the relationship between diet and brain health can be described as one of resilience, adaptability, and interconnectedness. This narrative extends beyond the reductionist view of nutrients and dietary patterns, integrating cultural, lifestyle, and molecular elements into a comprehensive account of cognitive resilience. As the chapters of research continue to unfold, the symphony of dietary strategies in neuroprotection continues to play, promising a harmonious and resilient cognitive landscape for individuals and societies alike. The nexus of nutrition and neuroscience, in its complexity and diversity, serves as an exemplar of the potential for transformative interventions that transcend the boundaries of conventional healthcare. The journey continues, and with each discovery, we approach a future in which nourishing the mind is not merely a dietary consideration, but a societal imperative.

In this synthesis of nutrition and neuroscience, we posit that future dietary strategies will transcend mere sustenance, becoming integral components of holistic approaches to brain health. The potential for accessible, personalized, and evidence-based interventions offers a source of hope in the context of the growing challenges posed by neurodegenerative diseases. As the chapters of research continue to unfold, the story of diet and brain health remains to be written, offering the promise of a healthier and more resilient cognitive landscape for individuals and societies alike.
